# Tolerance of Douglas Fir Somatic Plantlets to Aluminum Stress: Biological, Cytological, and Mineral Studies

**DOI:** 10.3390/plants9040536

**Published:** 2020-04-21

**Authors:** Holm Amara, Marie-Anne Lelu-Walter, Vincent Gloaguen, Céline Faugeron-Girard

**Affiliations:** 1 Laboratoire Peirene (EA7500), Faculté des Sciences et Techniques, Université de Limoges, 123, avenue Albert Thomas, F-87060 Limoges CEDEX, France; holm.amara@unilim.fr (H.A.); vincent.gloaguen@unilim.fr (V.G.); 2 INRAE, ONF, BioForA, 2163 avenue de la Pomme de Pin, CS 40001 – Ardon, F-45075 Orléans, France; marie-anne.lelu-walter@inrae.fr

**Keywords:** aluminum, *Pseudotsuga menziesii* (Mirb.), tolerance

## Abstract

Aluminum (Al) is well known as a potent inhibitor of plant growth and development. It is notably present in soils in the soluble and bioavailable form Al^3+^ when the soil pH drops below 5. This situation is frequent, especially in softwood forests when litter decomposition is slow. In the present work, we studied the effects of Al^3+^ on the growth and development of Douglas fir plantlets. Somatic plantlets, regenerated via somatic embryogenesis, were grown in vitro on media supplemented with different concentrations of aluminum chloride (AlCl_3_): 0 µM, 200 µM, 500 µM. and 1 mM. We show that a concentration of 500 µM AlCl3 in medium significantly reduced root elongation (−21.8%), as well as stem growth (−14.6%). Also, a 25% reduction in dry mass of the plantlets was observed in presence of a concentration of 200 µM of AlCl3. Histological analysis of root tissues revealed significant damage, especially in conducting vessels. In addition, mineral cation content of plantlets was disturbed under Al exposure. More particularly, the Mg and K contents of needles and the Ca content of stems and needles were significantly reduced in presence of a concentration of 500 µM AlCl3 in the culture medium (−35.6%, −33.5%, −24%, and −34% respectively). However, all these damages appeared at relatively high Al concentrations when compared with other herbaceous species. This study shed light on the ability of Douglas fir in vitro plantlets to cope with the acid-driven toxicity of Al.

## 1. Introduction

Douglas fir (*Pseudotsuga menziesii* (Mirb.)) is a coniferous species native of the West Coast of North America and is one of the most important worldwide timber species. In Europe, it is frequently used to satisfy an increasing demand for its wood, which presents outstanding mechanical properties and durability. Therefore, this species has been largely planted since the mid-20th century [1]. However, in opposition to deciduous forests, large coniferous plantations might have deleterious effects on woodland, notably because the slow litter decomposition leads to an acidification of the soil, which, in turn, increases the mobility of some elements. Aluminum (Al), as it is often considered as the third most abundant element in the Earth’s crust, is particularly affected by this phenomenon. For example, insoluble aluminum hydroxides (e.g., Al(OH)_3_), mostly present in neutral soils, give rise to the highly soluble Al^3+^ free species when pH drops to 5 or below. In addition, other sources such as atmospheric deposition, rainfall, river runoff, and human-driven activities (such as the use of fertilizers or the combustion of fossil fuels giving rise to acid rains) may increase Al content of soils [2]. Al^3+^ is readily bioavailable but it is considered as nonessential for plant growth and might be highly toxic for plants. However, the harmful effects of Al^3+^ depend on the plant species. For example, the growth of corn roots is inhibited in presence of a concentration of 75 µM Al [3], while the growth of tea (*Camellia sinensis*) seedlings is only delayed when treated with a concentration of 500 µM of Al and more [4]. Simon et al. [5] showed that Al blocks the development of tomato plantlets at concentrations above 25 µM. Al concentrations as low as 10 µM slowed down the growth of the roots of *Pinus massoniana* seedlings grown under hydroponic conditions [6]. Therefore, Al could be considered as one of the major factors that impede plant growth and development in acidic soils, which represent up to 40% of the total arable land in the world. 

One of the major symptoms of Al stress in plants is the inhibition of root elongation, which occurs by the disruption of cell division [7]. Since Al^3+^ is highly reactive, it is able to act on the cell wall and the plasma membrane [8,9] where it impairs ion transport and leads to a poor nutrient balance [10]. Moreover, Al promotes oxidative damage, e.g., lipid peroxidation and DNA damage, through excessive generation of reactive oxygen species (ROS) [2,11]. Plants could mitigate Al toxicity via two strategies: Exclusion and intracellular mechanisms. Exclusion defense can be achieved by modification of the pH of the rhizosphere [12], the secretion of organic acids having a chelating ability for Al^3+^ [13]. The trapping of Al^3+^ within the cell wall also helps the cells to escape from Al toxicity [7]. Intracellular mechanisms include accumulation of a wide array of antioxidant metabolites (proline, polyphenols, tocopherols) and enzymes (peroxidase (POX), superoxide dismutase (SOD), etc.) [13,14].

If the literature about the presence of Al in soil is abundant, studies often address the consequences of acid rains on the release of Al in soil [15,16,17], but more rarely address the tolerance of trees to Al. Some plant species seem to adapt to acidic soils and to the presence of Al^3+^, for example, coniferous species, which are largely cultivated in such conditions. Douglas fir trees are apparently unaffected by Al stress [18]. However, little is known about their capacity to tolerate high Al concentrations in soil solution. Keltjens et al. [19] have shown that the viability of one-year-old Douglas-fir plants was affected when transferred to an hydroponic culture system in the presence of concentrations of AlCl_3_ higher than 15 mg/L (equivalent to 112 µM) and that root growth was hampered by a concentration of 8 mg/L AlCl_3_ (60 µM) [20]_._ However, the mechanisms involved in the resistance to Al stress are currently unknown. In order to make progress in the understanding of the tolerance of Douglas fir trees to Al, we designed an in vitro study on Douglas fir plantlets obtained from somatic embryos, named somatic plantlets. This strategy offers several advantages, particularly controlled growth conditions (notably the Al concentration in the growth medium), a homogeneous genetic pattern, the avoidance of genetic variations, and the production of a large number of somatic plantlets regardless of seasonal variations. Also, and unlike seedlings obtained after sowing in soil, this method allows a direct and easy monitoring of root growth. This work was aimed at determining the tolerance level of Douglas fir somatic plantlets growing since their germination in the presence of increasing concentrations of Al. In the case of Douglas fir, a forest species of economic interest, our work describes, for the first time, the Al impact on growth by the means of phenotypic observations (root and stem length, number of needles), along with micromorphological and histological analysis of the root apices. The mineral status of Al-exposed plantlets has also been characterized, along with the distribution of Al in somatic plantlets.

## 2. Results

### 2.1. Effect of Al on the Growth of Douglas Fir Somatic Plantlets 

**Figure 1:**
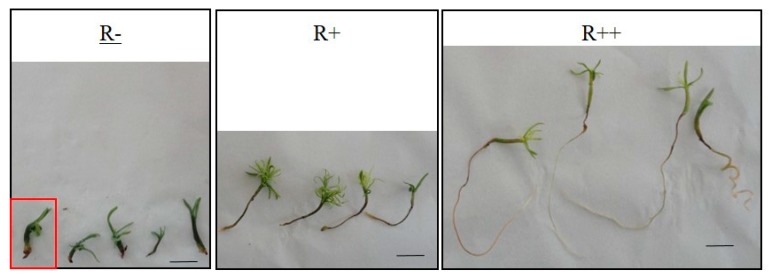
Douglas fir somatic plantlets grown on germination medium without addition of AlCl_3_ (control conditions) for eight weeks. Red box: Plantlet showing hyperhydricity symptoms. Bar: 1 cm.

One week after germination, plantlets were transferred on fresh germination media supplemented with different concentrations of AlCl_3_ (up to 1 mM) and incubated for an additional eight weeks. On control medium (i.e., without added AlCl_3_), Douglas fir plantlets presented different types of morphology (Figure 1). Three categories of plantlets could be defined depending on the length of the roots. The first one, called “R−”, characterized by an absence of root (Figure 1, R−), represented about 40% of the population; the second category, “R+”, with roots less than 40-mm long, accounted for about 30% of the population; and the last category, called “R++”, presented longer roots (length ≥ 40 mm), and totaled about 30% of the population. In addition to the absence of root in the R− category, several anomalies were identified in the R+ group, such as symptoms of hyperhydricity of the needles and hardening of roots and stems (red box, Figure 1). For these reasons, further experiments and assays have only been conducted on plantlets belonging to the R++ category.

**Figure 2:**
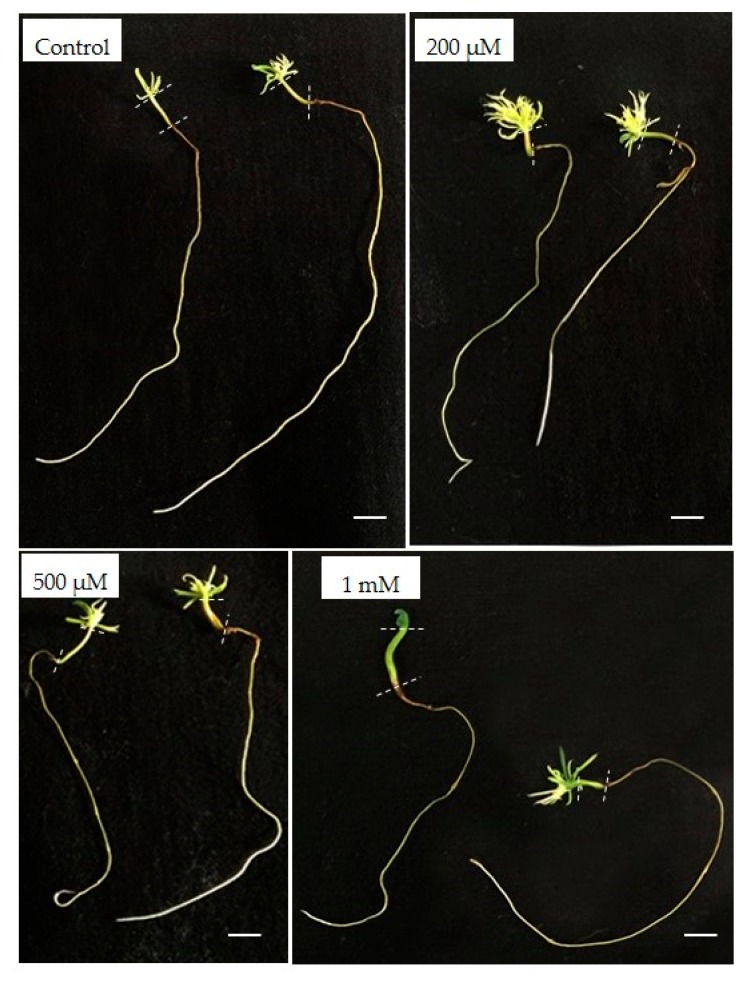
Effect of Al concentration on the morphology of 8-week-old Douglas fir somatic plantlets. Bar: 1 cm.

**Figure 3:**
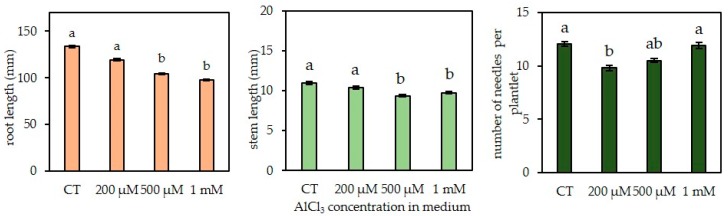
Effect of AlCl_3_ concentration in culture medium (range: 0–1 mM) on root length and aerial growth of Douglas fir somatic plantlets R ++ in vitro. CT: Control treatment, no addition of AlCl_3_ in the medium. Lengths of roots and stems were measured directly after harvest and the needles were counted. Each bar of the histograms represents the average and standard error (SE) of 13 independent experiments, each with 20 somatic plantlets. Different letters indicate significant differences between exposure conditions (*p* < 0.05), KRUSKAL test, n = 260 (mean +/− SE). R++ plantlets showed a root length > 40 mm after one week of growth on germination medium.

The presence of AlCl_3_, at a concentration as high as 100 µM in the germination medium, did not affect root or stem growth as shown by the phenotypic observations (supplementary data Figure S2). However, a significant inhibitory effect on root growth was observed in presence of a concentration 500 µM AlCl_3_. The average length of root was significantly reduced from 133 mm on the control medium to 104 mm (−21.8%) in the presence of a concentration of 500 µM AlCl_3_ (Figure 2 and Figure 3). Also, a significant decrease in stem length was observed in presence of a concentration of 500 µM of AlCl_3_ (−14.6%) as compared with the control. The average number of needles (12.04 needles per plant in control conditions) significantly dropped in presence of a concentration of 200 µM AlCl_3_ (9.8 needles per plant; −18.6%) but, surprisingly, an opposite trend was recorded with higher concentrations. Finally, in presence of a concentration of 1 mM AlCl_3_, this number was similar to what was seen in control conditions. It can be noticed that in the presence of a concentration of 1 mM of AlCl_3_, essentially, two phenotypes appeared: plantlets with a normal aerial part development (several needles) and plantlets with large and dark-green cotyledons but apparently devoid of developed needles (Figure 2). 

**Figure 4:**
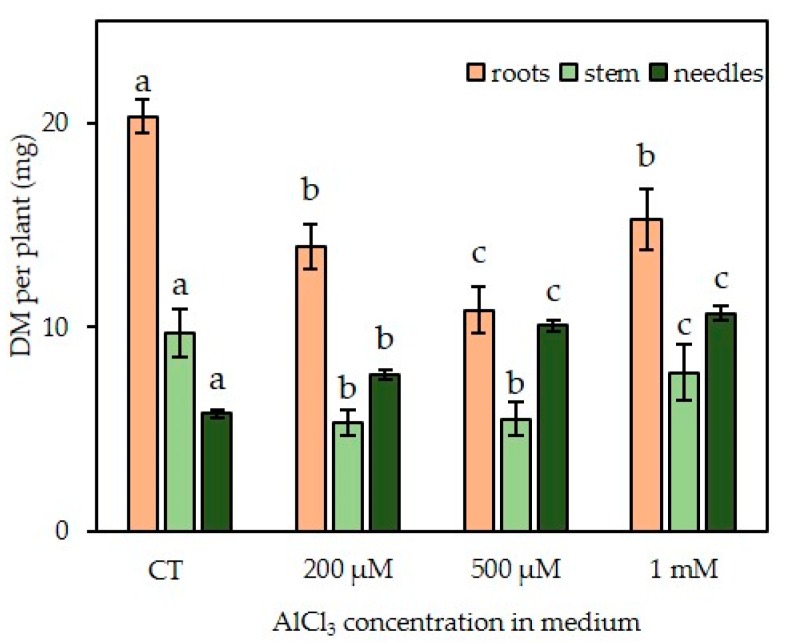
Effect of AlCl_3_ concentration (range: 0–1 mM) in culture medium on the average dry mass (DM) of various organs of in vitro-grown R++ Douglas fir somatic plantlets. For each harvest, the same organs of all somatic plantlets grown in the same condition were gathered for better accuracy. Organs were then lyophilized and weighed. Each bar of the histogram represents the mean and standard deviation (SD) of 13 independent experiments, each with 20 plantlets. Different letters indicate significant differences between exposure conditions (*p* < 0.05), ANOVA test. n = 13 (mean +/− SD). R++ plantlets showed a root length > 40 mm after one week of growth on germination medium. CT: Control treatment.

The average dry mass of the roots (Figure 4) decreased by approximately 31% in the presence of a concentration of 200 μM AlCl_3_ (from 20.3 mg per plant in control condition to 13.9 mg for 200 µM), and 47% in presence of a concentration of 500 µM AlCl_3_ (10.8 mg) as compared with plantlets grown in control medium. No significant variation in dry weight was recorded in presence of concentrations of 10 µM, 25 µM, 50 µM, and 100 µM AlCl_3_ (supplementary data Figure S3; one-way ANNOVA *P* < 0.05). However, from 500 μM to 1 mM, dry weight increased again to a value of 15.3 mg to finally reach the value observed with a concentration of 200 µM AlCl_3_. With regard to the stems, the average dry mass significantly decreased by about 45% in the presence of concentrations of 200 µM and 500 µM AlCl_3_ (5.3 mg and 5.5 mg per plant, respectively). The average dry mass increased in the presence of a concentration of 1 mM AlCl_3_ (7.8 mg) but remained lower than the control level (9.7 mg). On the other hand, the average dry mass of the needles gradually increased with AlCl_3_ concentration, to reach, in the presence of a concentration of 1 mM AlCl_3_, almost twice the value observed in control conditions (10.7 mg as compared with 5.8 mg).

**Figure 5:**
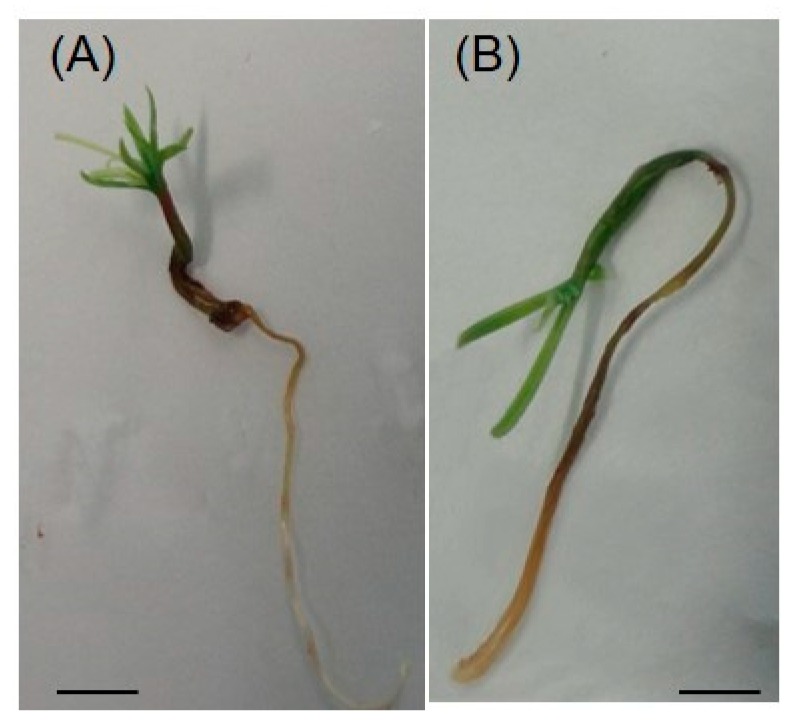
Symptoms of hyperhydricity of Douglas fir somatic plantlets cultured in vitro. (**A**) An R ++ plantlet grown in presence of a concentration of 500 µM AlCl_3_ having a normal appearance. (**B**) An R ++ plantlet at a concentration of 500 µM showing vitreous needles and stem. R++ plantlets showed a root length > 40 mm after one week of growth on germination medium. Bar: 1 cm.

The dry masses of the roots and the root elongation seemed to follow the same decline up to a concentration of 500 µM AlCl_3_. On the contrary, the dry mass of the needles gradually increased with AlCl_3_ concentration, whereas the average number of needles remained almost constant. This increase in mass may have been due to the hyperhydricity and thickening phenomenon encountered more and more frequently with higher concentrations in AlCl_3_: 20% of the plantlets showed hyperhydricity symptoms with 500 µM AlCl_3_ and 35% with 1 mM. In the latter case, needles appeared thicker, longer, and wider, with a trend to curl, and presented a dark-green color, along with being brittle and translucent. This phenomenon was also observed with the stems of 30% of the somatic plantlets grown in presence of a concentration of 1 mM AlCl_3_ (Figure 5).

### 2.2. SEM Observations of Root Tip Morphology

**Figure 6:**
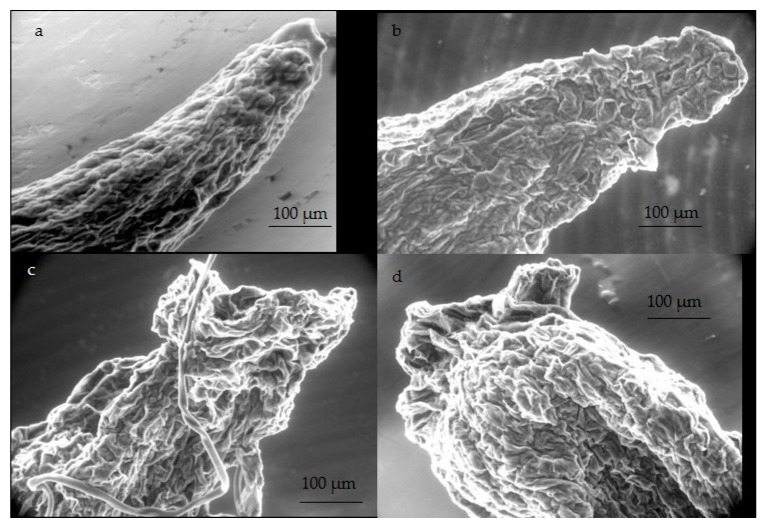
SEM observations of root tips of eight-week Douglas fir R ++ somatic plantlets grown in absence (**a**) or presence of concentrations of 200 µM (**b**), 500 µM (**c**), and 1 mM (**d**) AlCl_3_ in culture medium. Several samples have been observed, and the samples presented in this figure are representative of each one of the batches. R++ plantlets showed a root length > 40 mm after one week of growth on germination medium.

As the main phenotypic effect of Al toxicity concerned root growth, SEM observations of root tips were performed. Figure 6 shows alterations caused by an exposition to Al: the sharp, tapered shape of the apical zone of the control root (Figure 6a) led to a distorted and cracked ending in presence of a concentration of 1 mM AlCl_3_ (Figure 6c). Actually, the overall shape of the root apex was not significantly altered in presence of a concentration of 200 µM AlCl_3_ (Figure 6b). The root still presented a tapered tip, but started to make a small curl, which was more apparent in presence of a concentration of 500 µM AlCl_3_ (Figure 6c), along with presenting a rough surface.

**Figure 7:**
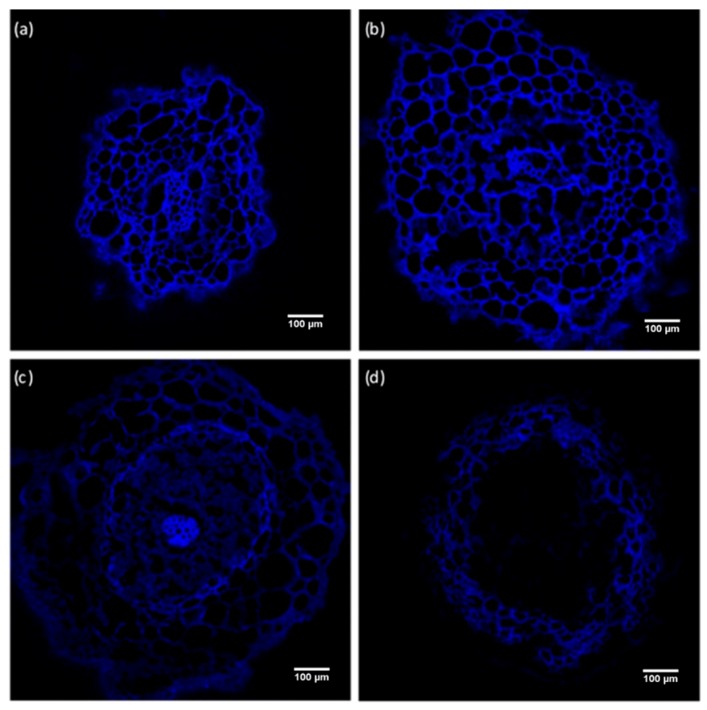
Confocal microscopy images of root cross sections of Douglas fir R ++ somatic plantlets at 10 mm of root tips, dyed with Calcofluor. Plantlets were grown for eight weeks in germination medium (**a:** Control), or in germination medium supplemented with different concentrations of AlCl_3_: (**b**) 200 µM, (**c**) 500 µM, (**d**) 1 mM. Several samples were observed, and the samples presented in this figure are each one representative of their batch. R++ plantlets showed a root length > 40 mm after one week of growth on germination medium.

Calcofluor staining allowed us to see the morphology of the cells, since this dye binds to cell wall polysaccharides. Confocal microscopy images showed that a distortion of the cellular structure of the root epidermis and cortex occurred in presence of AlCl_3_. The distortion increased with increasing concentration of AlCl_3_, as shown by the global decrease of fluorescence intensity of roots exposed to a concentration of 500 µM or 1 mM AlCl_3_ as compared with control conditions (Figure 7). In addition, Al led to damage of the cells of the conductive tissues of the central cylinder, as revealed by the partial disappearance of the fluorescent signal at the center of the root cross section in presence of a concentration of 1 mM AlCl_3_ (Figure 7d). 

### 2.3. Distribution of Al and Minerals in Different Organs of Douglas Fir Somatic Plantlets 

**Figure 8:**
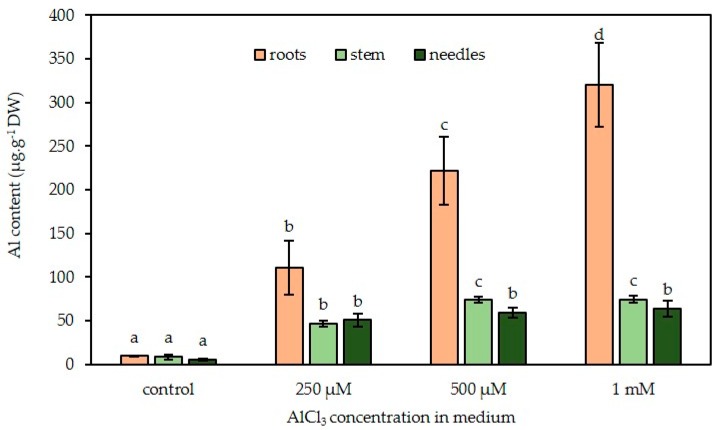
Al content (μg g*^−^*^1^ DW) in roots, stems, and needles of in vitro Douglas fir somatic plantlets exposed to concentrations of 0–1 mM AlCl_3_ in the culture medium. Each bar of the histogram represents the mean and standard deviation of three independent experiments, each with 10 plantlets per condition. Different letters indicate significant differences between exposure conditions (*p* < 0.05), ANOVA test. n = 4 (mean +/− SD). CT: Control treatment.

The presence of Al in Douglas fir plantlets was detected by microwave-induced plasma–atomic emission spectrophotometer (MP–AES) analysis. Al was already present in control somatic plantlets at 10 µg g*^−^*^1^ dry weight (DW), 7 µg g*^−^*^1^ DW, and 4 µg g*^−^*^1^ DW in roots, stems, and needles, respectively. Regardless of the concentration of Al in the culture medium, Al content of roots was always higher than the contents of the corresponding stems and needles. Al content decreased by a factor of approximately five in the aerial parts as compared with the roots. For example, at a concentration of 1 mM AlCl_3_ in the culture medium, roots contained 330 μg Al g*^−^*^1^ DW, while stems and needles contained 75 μg Al g*^−^*^1^ DW and 63 μg Al g*^−^*^1^ DW, respectively (Figure 8).

Also, it can be noticed that Al content of each organ increased with Al concentration in the germination medium. Al content of roots increased from 9.6 μg Al g*^−^*^1^ DW in control conditions to 320 μg Al g*^−^*^1^ DW in the presence of a concentration of 1 mM AlCl_3_. Under the same conditions, the stem contents varied from 9 μg Al g*^−^*^1^ DW to 74.7 μg Al g*^−^*^1^ DW and from 5.2 μg Al g*^−^*^1^ DW to 63.6 μg Al g*^−^*^1^ DW for the needles.

### 2.4. Localization of Al in the Roots of Douglas Fir Somatic Plantlets 

**Figure 9:**
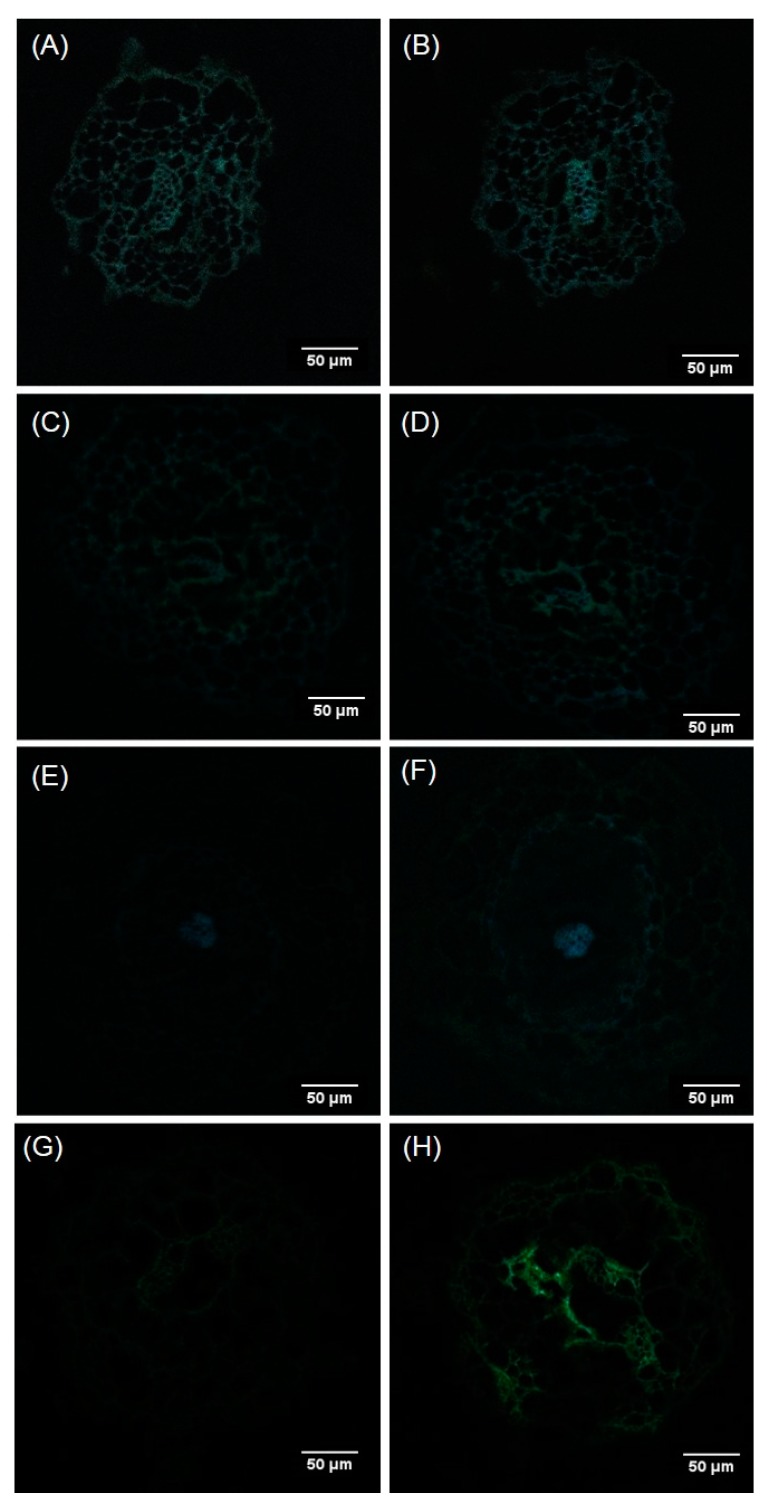
Al localization with Morin staining of root cross sections of Douglas fir somatic plantlets at 10 mm from the root tip. (**A**) Autofluorescence of plantlets grown under control conditions, or (**C**,**E**,**G**) in culture media supplemented with different concentrations of AlCl_3_ (200 µM, 500 µM and 1 mM, respectively). Fluorescence of Morin-stained samples: (**B)** Plantlets grown under control conditions, or (**D**,**F**,**H**) in culture media supplemented with different concentrations of AlCl_3_ (200 µM, 500 µM and 1 mM, respectively).

The localization of Al in root tissue was performed with confocal fluorescence microscopy on cross-sections of somatic plantlet roots after Morin staining. Dimming down the autofluorescence signal (AF) through spectral mode allowed us to evidence the fluorescence signal emitted by the Morin-Al^3+^ complex. This complex was detected even under the control conditions, although very faint at the level of the pith, more precisely in the cell walls of the conductive cells (Figure 9A). The signal emitted by this complex increased with increasing Al concentration in the culture medium. In presence of a concentration of 1 mM AlCl_3_, Al was located on almost the entire section, and associated to cell walls. Although signal intensities varied, fluorescence emission remained more pronounced at the level of cells of the vascular tissue and cortex (Figure 9H). 

### 2.5. Impact of Al on Mineral Content of Douglas Fir Somatic Plantlets

**Figure 10:**
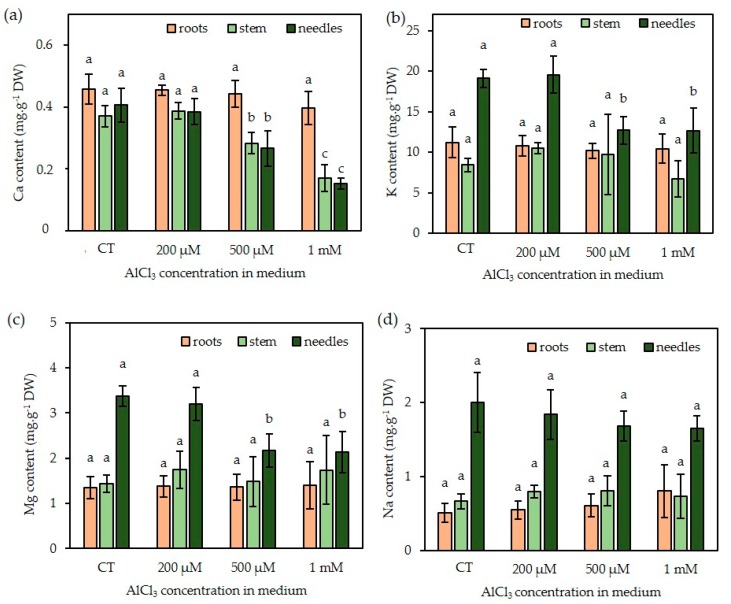
Mineral content of roots, stems and needles of Douglas fir somatic plantlets exposed to concentrations of 0–1 mM AlCl_3_. (**a**): Ca, (**b**): K, (**c**): Mg, (**d**): Na (contents expressed in mg g*^−^*^1^ DW). Each bar of the histogram represents the mean and standard deviation of four independent experiments, each with 10 plantlets. Different letters indicate significant differences between exposure conditions (*p* < 0.05), ANOVA test. n = 4 (mean +/− SD).

As mineral nutrition could be impaired by Al, some essential mineral nutrient contents were determined (Figure 10). 

The presence of Al did not seem to significantly affect Ca, K, Mg, and Na contents of the roots despite a tendency of increase in Na and a decrease in Ca for the highest Al concentration. On the contrary, in aerial parts, Ca content was significantly reduced in the shoots and needles in the presence of a concentration of 500 µM AlCl3. This parameter seemed to be the most affected by Al treatment, since the decrease in Mg and K was observed only in the needles in presence of a concentration of 500 µM AlCl3. Finally, Na content was not affected by the presence of Al in the medium in any part of the plants.

## 3. Discussion

Reports on Al toxicity in herbaceous species and crop plants are largely present in the literature, but only few studies have been done to determine the toxicity levels of Al for gymnosperms [6,21,22,23,24]. However, softwood forests are known to occupy areas where the acidic characteristic of the soil triggers the release of Al^3+^ in the soil solution; Al^3+^ is readily bioavailable for vegetation. Douglas fir is one of the most widely cultured resinous trees, but to our knowledge, its tolerance to Al is not well documented [19,20]. In this study, the tolerance of Douglas fir somatic plantlets was studied under controlled conditions. Somatic plantlets were obtained from somatic embryogenesis in order to benefit from a uniform genetic background. Also, in vitro conditions provided homogenous culture conditions, allowing us to individually test the effects of defined concentrations of AlCl_3_ in the culture medium. First, the biological material was chosen in function of its capacity to develop normal roots, e.g., more than 40-mm long, one week after transfer on the germination medium (R++). Indeed, some somatic plantlets did not show any root growth (called R−) despite their development of aerial parts, or had a very short root (< 4 mm: R+). So far, this observation had not been reported for Douglas fir somatic plantlets [25]. Zhao et al. [26] already described the absence of roots in American ginseng (*Panax quinquefolius*) somatic embryos germinated for six weeks with a rate of 50%. This anomaly has also been mentioned in the case of hybrid larch as one of the characteristics of early germination and as the result of a rapid transition from the embryonic stage to the germinal stage without a sufficiently long maturation phase [27]. This phenomenon could be attenuated, but not completely eliminated, with the addition of 40 μM or 60 μM abscisic acid to the germination medium of hybrid larch [28] or with the addition of active charcoal in the germination medium of American ginseng [29]. Consequently, we had to select somatic plantlets after one week of germination medium in order to obtain a very homogenous plant material for our experiments. Also, one-week-old somatic plantlets presenting hyperhydricity symptoms were discarded (vitreous aspects, thick stem or needles). 

When exposed to AlCl_3_, the growth and development Douglas fir somatic plantlets showed no alteration up to a concentration of 100 µM. However, in the presence of a concentration of 200 µM and higher concentrations of AlCl_3,_ we observed significant drops in root and stem dry mass, along with a decline in the number of needles. This result is similar to the study on *Pinus thumbergii* seedlings that exhibited no significant reduction in mass when treated with a concentration of less than 500 µM Al over 18 days [21]. Also, no effect on the plant growth of *Picea rubens* plantlets was observed when the concentration of Al was lower than 250 µM [24]. Nevertheless, the majority of the plant models described in the literature did not seem to withstand such high amounts of AlCl_3_. For example, the inhibition of root elongation of maize plantlets could be observed in the presence of AlCl_3_ concentrations in the culture medium as low as 25 μM [30]. Wheat root structure and growth were significantly altered when exposed to a concentration of 5 µM AlCl_3_ for four days [31]. A 40% inhibition of root growth was observed in red oak (*Quercus rubra*) in the presence of a concentration of 115 µM Al in hydroponic solution [32]. 

At the cellular level, the impairment or root growth is often caused by an alteration of the root apex and elongation zone. This impairment has presently been observed with Douglas fir somatic plantlets whose root apex was subjected to distortion and depression in presence of 200 µM and higher concentrations of AlCl_3_ in the culture medium, as shown by histochemical observations. The vascular cylinder seemed to be the most affected, since an important decrease in Calcofluor staining was observed in this part of the roots of plantlets exposed to a concentration of 1mM AlCl_3_. Al-triggered roughening of the surface of the root tip of plantlets is frequently described in the literature, as reported, for example, with in vitro seed germinations of *Brachiaria decumbens* exposed to a concentration of 200 μM AlCl_3_ [33]. Wagatsuma et al. [34] reported the various morphological changes in the root surface of pea seedlings observed by SEM after exposure to a concentration of 3 ppm AlCl_3_ (corresponding to 22.5 μM) during an in vitro seed culture. Deep transverse cracks in the cortex were visible, mainly in the middle part of the root at 40–70 mm from the root tip. In the same context, intense cellular disorganization has been reported in maize roots, particularly transverse breaks in the protodermal layers and the outer cortex at the end of the root [35]. It has been suggested that ruptures are the result of the increased root diameter due to a tearing of the cells with a ripping of the outer cortex and of the rhizodermal cells of the elongation zone [36,37,38]. According to these authors, inhibition of root elongation and root breakdown is the consequence of Al binding to cell wall components, leading to increased lignin biosynthesis and stiffening of the cell wall. In the present case, this phenomenon could explain the hardening of the Douglas fir roots, especially those exposed to a concentration of 1 mM AlCl_3_. Parenchyma cells of the cortex are responsible for the storage of the mineral nutrients. Therefore, the exposure to Al would affect the supply in nutrients and thus the plant growth and development. 

Concerning the Douglas fir needles, despite a reduction in their number, in the presence of a concentration of 200 µM and higher concentrations of AlCl_3_, their dry mass increased with increasing concentrations of Al in the culture medium. This result could be explained by the rising proportion of plantlets showing hyperhydricity symptoms during the eight-week exposure to Al, which reached about 30% in the presence of a concentration of 1 mM AlCl_3_. Usually, the hyperhydricity of in vitro-cultured plantlets is due to several factors such as the composition of culture medium, notably the concentration of phytohormones [39] and the type of gelling agent [40]. Van den Dries et al. [41] proposed that hyperhydricity is caused by a water logging of the apoplast, leading to an impairment of gas exchanges. Finally, this phenomenon could result in stress conditions that might be due to different factors, notably mineral nutrition disorders [42,43]. Here, we think that the Al-triggered hyperhydricity of Douglas fir somatic plantlets could be the consequence of an alteration of the inorganic nutrient balance.

Al exposure did not significantly change the contents of the major minerals in Douglas fir roots, although the Ca content seemed to decrease in presence of a concentration of 1 mM AlCl_3_. Aerial parts also showed a reduced Ca content (shoots and needles) in presence of 500 µM and 1 mM AlCl_3_. These trends have already been related in studies on wheat [44], and the authors also reported that Al^3+^ could displace Ca from homogalacturonan within the cell wall and could consequently reduce Ca uptake. We observed a drop in Mg^2+^ content of the needles in the presence of a concentration of 500 µM AlCl_3_. A similar result has been reported in a study about tea plants [45]. Also, van Praag et al. [46] have reported that a concentration of 1 mM AlCl_3_ exerted a much stronger inhibitory effect on the uptake and translocation of Mg in comparison with Ca. The reduced content in Mg might impair chlorophyll biosynthesis and, therefore, the photosynthetic apparatus, and finally the growth capacity of the plantlets. In the present study, the K content of the needles was reduced in the presence of a concentration of 500 µM AlCl_3_. In fact, controversial data concerning K has appeared in the literature, in which K^+^ uptake was said to be reduced in many tested plants in response to Al treatment [47], although an increase in K uptake has been reported in another study [48]. Na contents were similar in Al-treated somatic plantlets as compared with the control ones. Unlike Ca^2+^ Mg^2+^ or K^+^, Na^+^ is not essential for either growth or development. Hence, reduced growth-dependent Al toxicity will not alter Na level, as already suggested by Maathuis et al. [49]. Taken together, these results proved that structural and functional damage in the roots affects nutrient uptake, leading to reduced growth and mineral deficiency in Douglas fir somatic plants subjected to AlCl_3_ treatment at concentrations higher than 200 µM. This can also suggest an explanation for the needle hyperhydricity observed in presence of 500 µM and higher concentrations of AlCl_3_. Al intake by somatic plantlets disrupts the absorption of essential minerals, and this imbalance in mineral nutrition could be a major cause of malformation. Moreover, Singha et al. [50] showed that increasing Ca concentration in the culture medium reduced hyperhydricity. In the same context, Pasqualetto et al. [51] reported that a drop in K content led to a significant increase in the percentage of “vitrified” plants. 

Measurements of Al distribution in plantlets, as well as confocal microscopy of Morin-stained root cross-sections, revealed the presence of Al, although in low amounts, in plantlets grown in control conditions. This could be due to Al impurities in constituents of the culture medium, since MP-AES analysis of the control culture medium revealed the presence of a concentration of 4.63 µM Al. Similar results were observed in *Pinus massoniana* seedlings by the authors of [6] in control conditions, as roots, stems and needles contained about 23 µg Al g*^−^*^1^ DW, 18 µg Al g*^−^*^1^ DW, and 9 µg Al g*^−^*^1^ DW, respectively. In Douglas fir plantlets exposed to increasing concentrations of Al, roots appeared to be the richest part compared with the other organs (up to 320 µg g*^−^*^1^ DW for the highest Al concentration). This result is consistent with the fact that the roots are directly exposed to the culture medium. Nevertheless, part of the root-absorbed Al is actually transported to the stems and needles (74 µg g*^−^*^1^ DW and 63 µg g*^−^*^1^ DW, respectively, in the presence of a concentration of 1 mM AlCl_3_). The transfer and storage of Al in the aerial parts of the plant is a common feature of many tropical species. It is a coping strategy, or a response that helps the plant to survive to Al stress [52]. A typical example of a woody plant that accumulates large amounts of Al (>15 μg g*^−^*^1^) in its leaves is *Richeria grandis* (Euphorbiaceae, dicotyledon). This result was obtained with mature leaves taken from trees planted on an Al-rich soil [53]. Al content in scots pine needles steadily increased with increasing external Al and reached values of about 1000 mg kg*^−^*^1^ DW [19]. Moreover, the accumulation of Al in *Pinus massoniana* was mainly detected in roots (374 µg g*^−^*^1^), and only small amounts of Al were detected in the aerial parts (24.2 µg g*^−^*^1^ DW and 87.8 µg g*^−^*^1^ DW of stems and leaves, respectively) when treated with a concentration of 10 µM AlCl_3_ over 40 days [6]. These results are equivalent to the data herein reported.

Taken together, these results suggest that a concentration of 200 μM AlCl_3_ can be defined as the tolerance threshold for Douglas fir somatic plantlets, as higher concentrations lead to a drop in dry weight accompanied with a reduction in root elongation. This threshold value is equivalent to that causing significant reductions in root elongation of *Pinus thunbergii* seedlings after an 18 day-exposure to a concentration of (at least) 0.5 mM Al [21]. However, in comparison with tolerance thresholds of other plants such as maize [3] (25 µM), tobacco [54] (25 µM), wheat (7.5 µM) [55], and cowpea (30 µM) [47], Douglas fir somatic plantlets clearly showed a higher resistance to Al stress, similar to that of *Pinus thunbergii*, which leads us to suppose that gymnosperms could be more tolerant to Al than herbaceous species.

## 4. Materials and methods 

### 4.1. Plant Material and Growth Conditions

*Pseudotsuga menziesii* trees 4440 and 4474 originated from North Bend and Enumclaw (WA, USA), respectively. In 2012, controlled cross 4474 *×* 4440 (Orléans, France) was performed and somatic embryogenesis was induced from zygotic embryos giving rise to embryogenic lines [56]. Experiments were conducted with the highly embryogenic line TD15-1. Maturation of the somatic embryos was obtained according to Lelu-Walter et al. [25]. After eight weeks, cotyledonary somatic embryos (Figure S1) were isolated and transferred onto germination medium (pH 5.8) modified as follows: 3 g L*^−^*^1^ gellan gum (phytagel, Sigma), 2.5 mg L*^−^*^1^ nicotinic acid, 0.25 mg L*^−^*^1^ pyridoxin-HCl, and 2.5 mg L*^−^*^1^ thiamine-HCl. Petri dishes containing about 50 somatic embryos were kept at 24 °C in the dark for seven days and then placed for eight weeks under dim light, at a light intensity of 95 µmol m*^−^*^2^ s*^−^*^1^ (16-h photoperiod). Somatic embryos were considered germinated as soon as radicle emergence was observed. As soon as germinated somatic embryos developed their aerial part (epicotyl), they were considered as plantlets. 

### 4.2. Aluminum Treatment

Aluminum treatments were done by transferring Douglas fir somatic plantlets to germination media containing various concentrations of AlCl3 (AlCl3, 6 H20, Duchefa Biochemie): 0 μM, 200 μM, 500 µM, and 1 mM. After eight weeks, the somatic plantlets were harvested, and roots were carefully rinsed three times with deionized water. Biometric parameters (length of the root, length of the stem, number of needles, fresh mass of each compartment) were measured. Plantlets were then frozen and kept at −20 °C until analysis. Dry weights were measured on lyophilized samples.

### 4.3. Micromorphology Analysis of Root Tips by Scanning Electron Microscopy

To visualize the influence of Al supply on root surface structure, fresh roots were randomly selected from different Al conditions and analyzed by scanning electron microscopy (FEI, QUANTA FEG 450). 

### 4.4. Microscopic Examination of Roots 

#### 4.4.1. Calcofluor White Staining

To reveal the general morphology of root cells of Douglas fir somatic plantlets, root tissues were stained with Calcofluor White. This fluorochrome binds to cellulose in plant cell walls. Root samples consisting in 10-µm cross-sections, cut at a distance of 10 mm to 15 mm from the root tip, were stained with 0.02% Calcofluor White solution (Sigma-Aldrich, St. Louis) for 5 min at room temperature. The sections were then rinsed with distilled water. Samples were observed by laser confocal microscopy (LSM 510-META, ZEISS) (excitation: 254 nm; emission: 430 nm).

#### 4.4.2. Morin Staining

Morin staining was used to reveal the localization of Al in root tissue [57]. A 100-μM solution of Morin (Sigma-Aldrich) in 5 mM of ammonium acetate buffer pH 5 was used for the staining of Al in plant tissues. First, 10-µm-thick sections were rinsed with 5 mM of ammonium acetate buffer pH 5, then stained with Morin solution for 60 min and finally rinsed with buffer and deionized water for 5 min each. Sections (marked or not) were mounted in a few drops of Mowiol solution. The Al-Morin complex fluorescence emission was observed with a laser confocal microscope (excitation: 420 nm; emission: 510 nm). Spectral mode was used to check autofluorescence on control roots with and without staining, and Al-treated roots without staining (LSM 510-META, ZEISS).

### 4.5. Mineral Content and Al Quantification 

Samples of roots, stems, and needles from somatic plantlets were first lyophilized and ground. Then, for each sample, the plant powder (200 mg) was mixed with 2 mL of nitric acid (68%, Normapur) and 1 mL of hydrochloric acid (37%, Normapur) in a reactor according to Astier et al. [58] and then subjected to microwave digestion (Mars One, CEM) using a two phase-program: A ramp from 0 °C to 200 °C in 20 min followed by a 10-min plateau at 200 °C before cooling. The final volume was adjusted to 20 mL with ultrapure water. Al, Ca, K, Mg, and Na were then quantified using a microwave plasma-atomic emission spectrometer at 396.2 nm, 422.6 nm, 769.9 nm, 285.2 nm, and 589.5 nm, respectively (4100 MP-AES, Agilent Technologies).

### 4.6. Statistical Analysis

Biological experiments were performed at least three times independently and results were expressed as mean ±standard deviation (SD). Statistical analysis consisted of one-way ANOVA test using the PAST free software (Version 2.17c).

Biometric measurements of root length and aerial growth of Douglas fir somatic plantlets were performed on 260 plantlets independently and results were expressed as mean ± standard error (SE). As the data was not normally distributed, statistical analysis was tested with a nonparametric test of one-way ANOVA, the Kruskal–Wallis test, using the R software (Version 3.6.2.).

## 5. Conclusions

This study showed, for the first time, that in vitro-grown Douglas fir somatic plantlets, exposed since germination to Al stress, tolerate relatively high Al concentration in the culture medium in comparison with crop species. Indeed, a concentration of 200 µM AlCl3 was the threshold value to observe a drop in needle development and reduction in the dry mass of all plant parts. These impairments of plant development could be explained by a perturbation of mineral nutrition. Notably, Ca and Mg contents were reduced in aerial parts. Al could possibly disturb the supply in Mg, leading to a lesser photosynthetic activity and to the displacement of Ca from the cell wall, potentially causing alterations of mechanical properties and finally a collapse in tissue structure, as evidenced in root apex. Additionally, damages of the conductive cells of root could explain mineral disorders. The mechanism by which Douglas fir somatic plantlets could tolerate such high Al concentrations is currently under investigation, notably focusing on the cell wall modifications, as this compartment could act as a barrier against toxic elements, but also because it constitutes the main component of the wood, the most valorized part of the tree. Further investigations will be conducted on young Douglas fir trees grown on acidic forest soils (with high level of bioavailable Al3+) to validate the observations made here in vitro on plantlets and to determine the impact of Al on the building and the biochemical composition of Douglas fir wood.

## Supplementary Materials

The following documents are available online at https://www.mdpi.com/2223-7747/9/4/536/s1, Figure S1: Cotyledonary somatic embryos of Douglas-fir after 8 weeks of maturation, Figure S2: Effect of AlCl_3_ concentration in culture medium (range 0-100 µM AlCl_3_) on root and aerial growth of Douglas fir somatic plantlets in vitro, Figure S3: Effect of AlCl_3_ concentration in culture medium (range 0-100 µM) on the average dry mass of various organs of in vitro Douglas fir somatic plantlets R ++.
