# Supplementary material for: Tolerance of Douglas Fir Somatic Plantlets to Aluminum Stress: Biological, Cytological, and Mineral Studies
Source: Plants (Basel). 2020 Apr 21;9(4):536. doi: 10.3390/plants9040536 (PMC7238193; doi:10.3390/plants9040536)
Supplement: Supplementary file 1 [file plants-09-00536-s001.pdf]

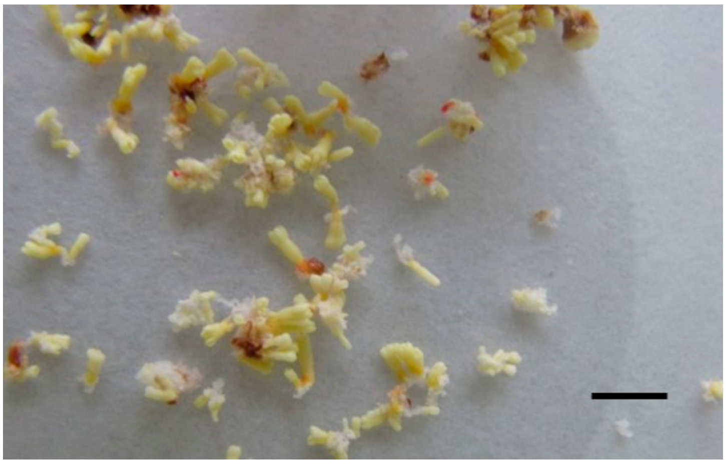

Figure S1: Cotyledonary somatic embryos of Douglas-fir after 8 weeks of maturation (bar = 3 mm).

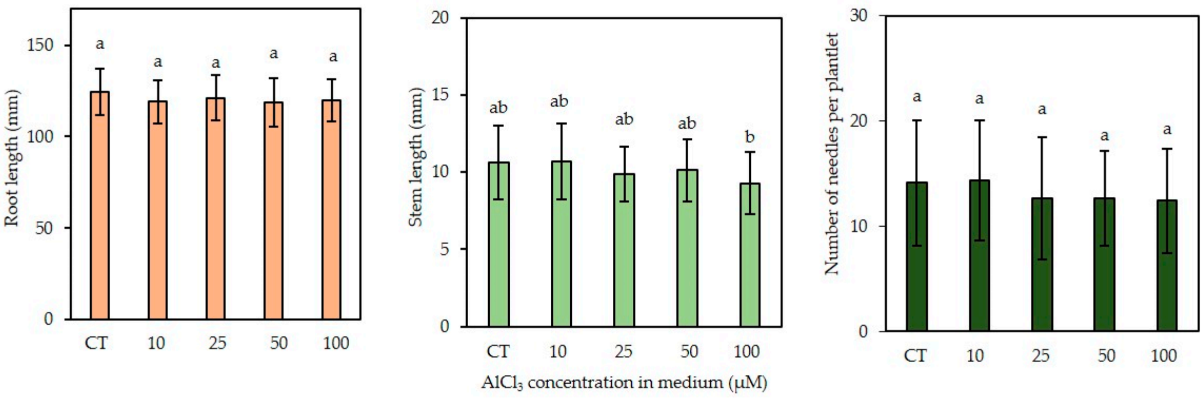

Figure S2: Effect of $AlCl_3$ concentration in culture medium (range 0-100 µM $AlCl_3$) on root and aerial growth of Douglas fir somatic plantlets *in vitro*. After one week on germination medium, somatic plantlets were transferred on Al-containing medium. Eight weeks later, plantlets with a root length of at least 40 mm (R ++) were kept and analyzed. Directly after harvest, the length of the roots and stems and the number of needles were measured. Each bar of the histogram represents the average and standard deviation (SD) of four independent experiments with each 20 somatic plantlets per condition. Different letters indicate significant differences between exposure conditions ($p < 0.05$), ANOVA test, n = 80 (mean +/- SD). CT: control treatment.

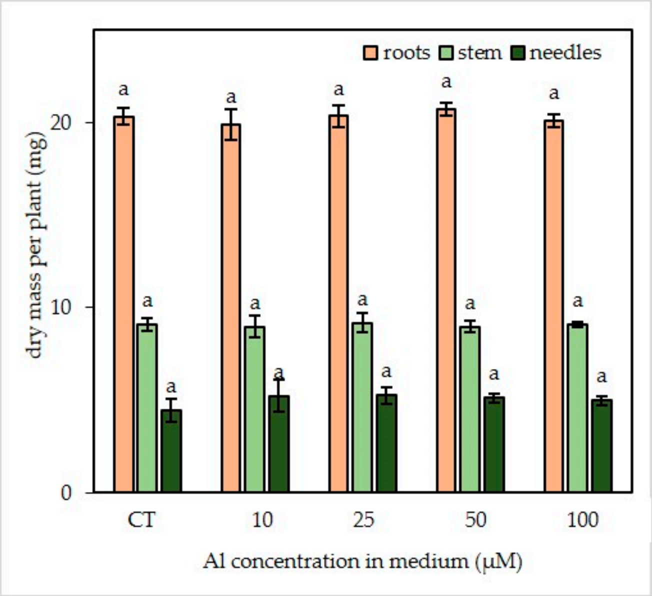

Figure S3: Effect of $AlCl_3$ concentration in culture medium (range 0-100 µM) on the average dry mass of various organs of *in vitro* Douglas fir somatic plantlets R ++. For one harvest, the same organ of all somatic plantlets of the same condition were grouped to obtain a more accurate average mass. Organs were then lyophilized and weighed. Each bar of the histogram represents the mean and standard deviation (SD) of four independent experiments with each 20 plantlets per condition. Different letters indicate significant differences between exposure conditions ($p < 0.05$), ANOVA test. n = 4 (mean +/- SD). R++ plantlets showed a root length > 40 mm after one week of growth on germination medium. CT: control treatment.
